# Emotional–cognitive integration in aging: the role of alexithymia in mild cognitive impairment

**DOI:** 10.3389/fpubh.2026.1737901

**Published:** 2026-03-19

**Authors:** Giulia Marselli, Ilaria Corbo, Anna Pecchinenda, Giuseppe Forte, Francesca Favieri, Giovanna Troisi, Barbara Blasutto, Maria Casagrande

**Affiliations:** 1 Department of Dynamic and Clinical Psychology and Health Studies, Sapienza University of Rome, Rome, Italy; 2 Department of Psychology, Sapienza University of Rome, Rome, Italy; 3 Department of Psychology, University “G. d’Annunzio” of Chieti and Pescara, Chieti, Italy; 4 Department of Experimental Psychology and Mind, Brain and Behavior Research Center (CIMCYC), University of Granada, Granada, Spain

**Keywords:** aging, alexithymia, cognitive functions, emotional awareness, emotion–cognition interaction, externally oriented thinking, mild cognitive impairment

## Abstract

**Introduction:**

Aging is accompanied by a range of cognitive and emotional changes. Among these, difficulty in identifying and describing feelings and a tendency toward externally oriented thinking have been associated with frank cognitive decline. This pattern is known as alexithymia and reflects emotional dysregulation. *Research Questions–*This study aimed to investigate the largely unexplored relationship between alexithymia and cognitive functioning in older adults within the context of mild cognitive impairment (MCI).

**Methods:**

Three hundred and twenty adults aged 50–80 years classified as healthy controls, amnestic MCI (aMCI), or non-amnestic MCI (naMCI), completed a comprehensive neuropsychological assessment and the 20-item Toronto Alexithymia Scale (TAS-20).

**Results and discussion:**

Participants with aMCI showed significantly higher levels of alexithymia, compared to healthy controls. This pattern suggests that emotional dysregulation is more pronounced in individuals with memory-related cognitive decline. In both the aMCI and naMCI groups, correlations between alexithymia scores and cognitive measures were negative, indicating that higher alexithymia was associated with poorer cognitive performance. In contrast, these associations were weak in healthy controls, implying that the link between emotional processing difficulties and cognitive inefficiency emerges primarily in MCI. Taken together, these findings point to a specific interplay between emotional and cognitive domains in the early stages of neurodegenerative decline. Accordingly, elevated alexithymia in aMCI individuals might represent a socio-emotional marker of prodromal Alzheimer’s disease, highlighting the importance of considering emotional regulation in the assessment of cognitive aging.

## Introduction

Aging is a complex and multifaceted process that goes far beyond the physical changes of later life. It involves a gradual transformation of cognitive and neural systems that sustain attention, memory, executive functions, and mental representation (1). Alongside these cognitive changes, emotional functioning also evolves; importantly, evidence from daily-life studies and broader reviews suggests that emotional regulation and psychological flexibility are often preserved—or even improved—in healthy aging (2). Accordingly, reduced emotional flexibility may be more characteristic of pathological aging and emerging neurocognitive decline than of aging per se (3).

Considering the societal impact of a rapidly growing older population, the World Health Organization has identified detection of pathological aging as a major priority. This includes not only delaying neurodegenerative processes but also fostering emotional well-being through active and meaningful aging (4). In an attempt to overcome this challenge, much research is focusing on Mild Cognitive Impairment (MCI), a prodromal risk-state clinical condition considered a transitional phase between physiological aging and dementia, particularly Alzheimer’s disease (5, 6) and on possible interventions to prevent the progression to dementia [see (7)]. More specifically, Mild Cognitive Impairment is defined by subjective and objective cognitive deficits that exceed age-related changes but do not yet interfere with daily functioning and remain below the threshold for dementia. MCI can manifest as amnestic MCI (aMCI), primarily affecting memory, or non-amnestic MCI (naMCI), which involves other cognitive domains. Each subtype can further present as single-domain or multiple-domain, reflecting the degree of cognitive compromise (5).

Over the past decade, attention has shifted from viewing MCI as a purely cognitive condition to recognizing it as a state that also affects emotional and social cognition. Abilities such as theory of mind (ToM) and mentalizing, the capacity to infer and represent others’ thoughts, intentions, and emotions, have been shown to decline with age and even more so in neurodegenerative conditions (8). These functions are fundamental for empathy, social adaptation, and emotional understanding, and their deterioration can profoundly alter interpersonal relationships and self-awareness. Empirical studies have provided converging evidence for early socio-cognitive changes in dementia (8) and MCI, especially in aMCI (9, 10). Although findings in aMCI remain mixed. More specifically, Baglio et al. (11) provided evidence that ToM undergoes both behavioral and neural alterations in individuals with aMCI. Their study revealed that aMCI patients performed worse on second-order false belief tasks, indicating ToM decay at the behavioral level, and showed reduced activation in parts of the ToM neural circuit (e.g., superior temporal sulcus and temporal pole). However, preserved activity in frontal regions and mirror neuron systems (e.g., Broca’s area and precentral gyrus) appeared to compensate for these deficits, maintaining good performance in tasks such as the Reading the Mind in the Eyes test. These findings suggest that even subtle disruptions in mentalizing networks may emerge early in aMCI, preceding more profound ToM decline.

In addition, Quaranta et al. (12) explored empathy in individuals with MCI due to Alzheimer’s disease and found reduced Perspective Taking and Fantasy, alongside increased Empathic Concern. Structural MRI analyses linked these changes to alterations in temporoparietal regions critical for social cognition, suggesting that deficits in empathy regulation and ToM processing may share common neurobiological underpinnings. These early empathic alterations, possibly reflecting diminished self-regulation and mental flexibility, are in keep with the hypothesis that socio-cognitive dysfunction occurs before overt dementia.

Moreover, Wang et al. (13) demonstrated that attentional mechanisms for emotional stimuli are already disrupted in MCI as well as in AD patients. Using event-related potentials (ERPs), they showed delayed P3 latencies and hypoactivation in frontal and temporal regions under emotional conditions, particularly happiness and sadness. These electrophysiological changes highlight that alterations in emotional attention and processing represent another early signature of neurocognitive decline in MCI.

Longitudinal data also indicate that both affective and cognitive components of ToM tend to decline in aMCI, with affective ToM emerging as a residual capacity that may initially compensate for cognitive decline (14). Recent studies also suggest that ToM decline may already be present in aMCI, even before the onset of dementia (14). Such socio-cognitive alterations point to an early disruption in the ability to process and integrate emotional information, which may not be limited to understanding others’ emotions but may also extend to recognizing and describing one’s own emotional states. Importantly, the capacity to accurately interpret others’ emotions presupposes an adequate level of self-awareness and introspective access to one’s own affective states (15). Self-related emotional awareness therefore represents a foundational component of social cognition, supporting processes such as mentalizing, empathy, and Theory of Mind. A shared disruption of these integrated self- and other-related socio-emotional processes may emerge early in neurodegenerative conditions. Within this framework, alexithymia—characterized by difficulties in identifying, describing, and reflecting upon one’s own emotions—represents a particularly relevant construct for understanding early affective alterations in cognitive aging and MCI.

Alexithymia refers to difficulties in identifying, describing, and processing one’s own emotions. It is characterized by a thinking style with a preference for external details and factual thinking [externally oriented thinking; (16)]. The prevalence of alexithymia in general population ranges between 7.1% (17), 10% (18), and 12.8% (19). Higher levels of alexithymia are associated to restricted capacity to fantasize and describe physical symptoms rather than affective states (20, 21). The main features of alexithymia include difficulty describing emotions verbally, a scarcity of inner fantasy life, concrete and externally focused speech and thought, reliance on physical symptoms as expressions of affective states, and difficulty in using bodily sensations as emotional signals (21, 22). It has been suggested that from about 30 years of age, the process of aging is associated with higher levels of alexithymia (23), and that the link between cognitive dysfunction and alexithymia should be apparent across the lifespan [e.g., (23)]. More specifically, there is evidence that alexithymia contributes to deficits in learning and memory, especially for emotional—but not for neutral information and contexts (24, 25). Although, cross-sectional evidence points to an association between alexithymia and poor cognitive functions, which is not specific to emotional information. That is, Correro (26) assessed the relationships between alexithymia, memory and executive functions in healthy young and old adults, showing that alexithymia contributed to poorer memory and executive functions in both age groups. This evidence suggests that alexithymia is a risk factor for age-related cognitive decline. Other studies have specifically investigated the relationship between alexithymia and MCI in aging. For example, Smirni et al. (27) found that individuals with MCI reported higher alexithymia levels compared to healthy controls, with negative correlations between alexithymia scores (Toronto Alexithymia Scale-20—TAS-20—total and the “difficulty identifying feelings” factor) and cognitive functions such as long-term verbal memory and working memory. Similarly, Yuruyen et al. (28) observed alexithymia traits in individuals with MCI and in individuals with mild Alzheimer’s disease. Whereas these findings indicate a potential link between cognitive impairment and difficulties in processing emotional information, their broader significance remains to be fully clarified (23).

This is because, besides the association between alexithymia and cognitive decline, alexithymia has also been associated with poorer processing of emotional information and reduced social cognition in healthy adults (29). Specifically, Demers and Koven (29) demonstrated that the externally oriented thought component of alexithymia is inversely related to affective ToM, suggesting that diminished mentalizing ability may underpin this aspect of emotional detachment. Recent evidence also suggests that alexithymia is linked not only to cognitive and emotional dysfunctions but also to physiological regulatory processes, such as circadian blood pressure variation, pointing to a close interplay between emotional regulation and autonomic functioning (30, 31).

Taken together, these findings portray a coherent picture: socio-cognitive functions such as ToM, empathy, and mentalizing begin to decline early in MCI, potentially leading to increased alexithymia, particularly in the externally oriented dimension. This pattern may reflect an emerging disconnection between emotional self-awareness and cognitive control, a hallmark of the early stages of Alzheimer’s disease. However, despite the growing recognition of emotional and cognitive dysfunction in aging, no prior studies have directly examined the specific relationship between alexithymia and cognitive impairment in aMCI populations. Establishing such a link could provide valuable insight into early socio-emotional markers of disruption in identifying one’s own feelings that accompany prodromal Alzheimer’s disease.

Accordingly, the present study investigates the relationship between alexithymia and cognitive impairment in older adults, with a particular focus on the amnestic subtype of MCI. Building on previous evidence, we hypothesize that individuals with aMCI will display higher levels of alexithymia than healthy control, especially in the externally oriented thought dimension, reflecting an early disconnection between cognitive and emotional self-processing that may signal the onset of Alzheimer’s pathology. Furthermore, we expect that higher alexithymia scores will be associated with poorer performance across cognitive domains within the MCI group, supporting the view that alexithymia reflects not merely a personality feature but an early socio-emotional marker of cognitive impairment.

## Method

We report how we determined our sample size, all data exclusions (if any) and all measures in the study.

### Participants

Three hundred and twenty adults voluntarily participated in the present study. Mean age was 61.7 years (SD = 7.9). Females represented 68% of the sample. The required number of participants was calculated using G*Power (Version 3.1.9.4). For between groups differences (MCI vs. Controls) on the TAS-20, with a *d* = 0.050, an alpha of 0.05, and a power of 0.95, a minimum of 63 participants per group is necessary. For the correlational analyses between alexithymia and global cognitive performance, with *r* = 0.30, a minimum of 85 is needed. In line with these *a priori* estimates, we aimed to recruit a sample larger than the minimum required to ensure adequate statistical power after stratifying the MCI sample into subtypes (aMCI and naMCI) and to increase the precision and stability of effect-size estimates. In addition, because clinically relevant alexithymia traits occur in a minority of the general population [≈7.1–12.8%; (17, 19)], a larger sample increases the likelihood of capturing sufficient variability across TAS-20 scores for the planned group comparisons and correlational analyses.

For our sample, schooling ranged from 5 to 31 years, with a mean number of 15.0 (SD = 3.9). The inclusion criteria were as follows: age over 50 years old, absence of psychiatric disorders (excluding anxiety and depression), absence of neurological diseases (excluding migraine and headaches), absence of cerebrovascular disorders, head trauma, brain injury, or brain surgery, and a score in the Mini-Mental State Examination greater than 23. We opted for including the lower age limit of 50 to capture early or prodromal stages of cognitive decline and to maximize variability in both cognitive functioning and emotional-processing traits. Mild cognitive impairment and subtle cognitive changes frequently emerge in midlife, therefore including adults aged 50–64 allows us to study alexithymia in relation to early cognitive changes that precede diagnosable dementia. This broader age range enhances the clinical relevance for prevention, reduces cohort and survivor biases that can arise from restricting samples to the oldest-old, and improves statistical power by increasing between-subject variability.

### Material

#### Anamnestic interview

A face-to-face structured interview collected general and medical information. Specifically, we gathered biographical information (age, marital status, schooling, occupation, etc.), lifestyle information (smoking, alcohol, and coffee consumption), psychological health information (including past and/or present psychotherapeutic treatments) and medical information (blood pressure values, cholesterol, triglycerides, blood glucose, familiarity with specific clinical pictures, past and/or actual medical problems, drug prescriptions).

#### Toronto alexithymia scale

TAS-20 [TAS-20; (32); Italian version: (33)] assessed alexithymia, through three sub-scales: difficulty identifying feelings (TAS F1), difficulty describing feelings (TAS F2), and externally oriented thinking (TAS F3). The Italian version of this questionnaire reported a Cronbach reliability coefficient of 0.75. Each item is scored on a 5-point Likert-type scale (1 = strongly disagree; 5 = strongly agree). Scores range from 20 to 100, with higher scores indicating the presence of alexithymia traits.

#### Neuropsychological assessment of MCI

##### Global cognitive functioning

a The Mini-Mental State Examination [MMSE; Italian Validation: (34, 35)] evaluates overall cognitive function by examining temporal and spatial orientation, short-term memory, attention, calculation, recall, language abilities, and praxis. The highest possible score is 30, with scores of 23 or below suggesting cognitive impairment, which may vary from mild to severe (36).
b The Raven’s Standard Progressive Matrices [RSPM; (37)] is a measure of logical-deductive reasoning used to evaluate fluid intelligence. It includes 60 matrices, organized into five sets of 12 items that progressively increase in difficulty. Each matrix displays a figure with a missing element and offers six to eight possible choices. Participants are required to select the option that best completes the figure. Scoring is based on the total number of correct responses, ranging from 0 to 60.
c The Activities of Daily Living [ADL; (38)] scale evaluates a person’s capacity to carry out basic self-care tasks. It comprises six items that measure independence in areas such as hygiene, mobility, feeding, and continence. Scores range from 0, indicating total dependence, to 6, reflecting full autonomy.
d The Instrumental Activities of Daily Living [IADL; (39)] scale assesses the ability to perform more complex tasks required for independent living. It includes eight items addressing functions such as meal preparation, shopping, housekeeping, laundry, transportation use, telephone use, medication management, and financial handling. Scores vary from 0 (complete dependence) to 8 (complete independence).

##### Memory

e The Digit Span Forward test (40) measures verbal short-term memory. Participants are required to repeat a sequence of digits in the exact order presented by the examiner. When a sequence is repeated correctly, a new sequence is given with one additional digit; if the attempt is incorrect, another sequence of the same length is administered. The test continues until two consecutive errors occur. The final score reflects the individual’s short-term memory span.
f Rey Auditory Verbal Learning Test (41) evaluates both immediate and delayed memory. Participants are presented with a list of 15 words, repeated five times, with immediate recall required after each presentation. Delayed recall is assessed once, following a 15-min interval during which participants perform visuospatial tasks. Scoring assigns 1 point for each correctly recalled word per trial, yielding an immediate recall total ranging from 0 to 75 and a maximum delayed recall score of 15.
g Babcock’s Tale (42) measures both short- and long-term semantic memory. The examiner reads a brief story, which the participant is asked to recall immediately. The story is then read once more, and recall is tested again after a 10-min delay filled with visuospatial activities. Each recall is scored on a scale of up to 8 points, based on the number of main events and details correctly remembered.
h Rey-Osterrieth Complex Figure–delayed recall [FRD; (43)] evaluates long-term visuospatial memory. Participants are first asked to replicate a complex line drawing by copying it freehand and after a 10-min interval to reproduce it from memory. The total score is based on the accuracy of the elements in both the copied and recalled drawings, considering their placement and fidelity.
i Immediate Visual Memory [IVM; (44)] measures short-term visuospatial memory. Participants view a figure for 3 s and must select the correct match from four options. The task includes 21 figures, with 1 point awarded for each correct response, resulting in a total score between 0 and 21.

##### Language

j Sentence construction (41) evaluates syntactic and verbal expression skills. Participants are presented with a set of words and are asked to construct a grammatically correct and meaningful sentence using them. Responses are scored based on accuracy, grammatical correctness, and coherence of the sentence. The total score reflects the participant’s ability to produce well-formed sentences, with higher scores indicating better verbal and syntactic performance.

##### Attention

k Visual Search Test (42) assess selective attention and visual scanning abilities. Participants are presented with a matrix of numbers or symbols and are asked to identify and mark target items within a limited time. Scoring is based on the number of correct targets identified, with higher scores indicating better attentional performance. Errors of omission or commission can also be recorded to provide additional insight into attention accuracy.
l Trail Making Test–A form [TMT-A; (45)] evaluates attention and processing speed. In this task, participants are required to connect a series of numbers [1–13] arranged randomly on a page in ascending order as quickly as possible. The score is based on the time, in seconds, needed to complete the task.

##### Visuospatial abilities

m Clock Drawing Test [CDT; (46)] evaluates visuospatial skills. Participants are first shown a blank circle and asked to place the numbers of a clock within it. They are then instructed to draw the clock hands to indicate a specific time (11:10).
n Rey-Osterrieth Complex Figure–copy [FRI; (43)] evaluates constructive praxis. Participants are first asked to replicate a complex line drawing by copying it freehand and later to reproduce it from memory after a 10-min interval. The total score is based on the accuracy of the elements in both the copied and recalled drawings, considering their placement and fidelity.
o Copying Drawings with and without programming elements [CD, CDP; (44)] valuates an individual’s praxis and visuospatial abilities. In the first part, the participant copies a presented figure (stimulus). In subsequent trials, figures containing some elements of the original stimulus, such as lines, dots, or angles, are shown, and the participant is asked to complete the missing parts and accurately reproduce the figure. Points are awarded based on performance.

##### Executive functions

p The Digit Span Backward test (40) is commonly used to assess working memory capacity and executive functions, particularly those involved in attention control, cognitive flexibility, and the manipulation of information held in short-term memory.
q Trail Making Test–B form [TMT-B; (45)] evaluates set-shifting ability, requiring participants to alternately connect numbers [1–13] and letters (A–N) as quickly as possible. The score reflects the total time, in seconds, needed to complete the task.
r Verbal fluency by phonemic (PF) and semantic (SF) categories (47). The phonemic verbal fluency test evaluates phonemic fluency, clustering, and task-switching abilities. Participants must generate as many words as possible in 1 min, with each word starting with a specific letter provided by the examiner. Three letters (e.g., L, F, P) are tested, giving the participant a total of 3 min. The score is the total number of valid words produced, excluding repetitions and proper nouns. The semantic verbal fluency task measures semantic verbal fluency, clustering, and task-switching. Participants are asked to produce as many words as possible within a given semantic category. Three categories (e.g., fruits, animals, car brands) are tested, with 1 min allowed per category. The score is the total number of correct words, excluding repetitions.

### Procedure

Participants were recruited through advertising (posters, web ads, word of mouth) and tested in the laboratory. Eligible participants signed the informed consent before completing the neuropsychological tests. The evaluation started with the anamnestic interview, then the tests and questionnaires were administered in randomized order. The assessment lasted approximately 3 h and was divided into two parts, separated by an interval. In some cases, the evaluation was completed on two different days. This study was conducted according to the Declaration of Helsinki and was approved by the Institutional Review Board of the Department of Psychology, Sapienza University of Rome (protocol number: 0001063).

### Group classification

In keep with Petersen’s criteria (5), the cut-off was set at minus 1.5 SD. Participants were classified as healthy controls (HC; N = 175; 66% female, mean age = 60.35, SD = 7.04, mean years of education = 15.85, SD = 3.62) and with MCI (N = 145; 69% female, mean age = 63.43, SD = 8.48, mean years of education = 13.99, SD = 4.06). Participants with MCI were further divided into two groups depending on the presence of an impairment in the memory domain: (a) amnesic MCI (aMCI; *N* = 64, 61% female, mean age = 65.75, SD = 8.19, mean years of education = 13.23, SD = 3.76) or preserved memory but impairments in other cognitive domains: (b) non-amnestic MCI (naMCI; *N* = 81, 80% female, mean age = 61.60, SD = 8.30, mean years of education = 14.58, SD = 4.21; see Table 1).

**Table 1:** Demographic characteristics of the sample according to Petersen’s criteria (5).

Group	*N*	Female (%)	Age, M (SD)	Education (years), M (SD)
Healthy controls (HC)	175	66%	60.35 (7.04)	15.85 (3.62)
Mild cognitive impairment (MCI)	145	69%	63.43 (8.48)	13.99 (4.06)
Amnestic MCI (aMCI)	64	61%	65.75 (8.19)	13.23 (3.76)
Non-amnestic MCI (naMCI)	81	80%	61.60 (8.30)	14.58 (4.21)

### Data analyses

A series of ANOVAs were conducted with Group (3: HC, aMCI, naMCI) as the independent variable and demographic (age, education), cognitive (MMSE and RSPM), psychological (GDS, BDI) functional (ADL and IADL) characteristics, and lifestyle habits (e.g., cigarettes, coffee, or alcohol consumption, number of children, and number of cohabitants) as dependent variables (for means and standard deviations see Table 2). When statistically significant differences in demographic variable were present, they were used as covariates in the statistical analyses. In addition, the *χ*^2^ test was used to evaluate whether the three groups differed on categorical variables (e.g., sex and marital status).

**Table 2:** Mean (±SD) of demographic variables according to the diagnosis.

Demographic variables	HC	aMCI	naMCI	*F*	*p*
	(*N* = 175)	(*N* = 64)	(*N* = 81)		
Age	60.3 (7.04)	65.8 (8.19)	61.6 (8.30)	11.8	<0.001
Years of education	15.9 (3.62)	13.2 (3.76)	14.6 (4.21)	11.8	<0.001
ADL	5.98 (0.13)	5.89 (0.44)	6.0 (0.0)	5.22	0.006
IADL	7.91 (0.43)	7.73 (0.65)	7.94 (0.33)	4.24	0.015
MMSE	29.3 (0.89)	28.1 (1.69)	28.8 (1.28)	25.3	<0.001
RSPM	37.2 (6.20)	30.9 (8.75)	32.3 (6.95)	25.6	<0.001
Cigarettes/day	2.63 (5.90)	2.85 (6.15)	2.69 (6.66)	0.03	0.99
Coffee/day	2.35 (1.69)	1.99 (1.30)	2.34 (1.59)	1.25	0.23
Alcohol/week	3.99 (9.11)	3.28 (4.76)	3.45 (4.87)	0.275	0.69
N. of children	1.59 (1.02)	1.97 (0.87)	1.81 (1.01)	3.78	0.024
N. of cohabitants	1.53 (1.37)	1.34 (1.24)	1.57 (1.43)	0.570	0.62
BDI	7.50 (7.59)	8.70 (9.23)	8.20 (8.19)	0.586	0.49

HC, healthy controls; aMCI: amnestic mild cognitive impairment; naMCI, non-amnestic mild cognitive impairment; ADL: Activities of Daily Living; IADL: Instrumental Activities of Daily Living; MMSE: Mini-Mental State Examination; RSPM: Raven’s Standard Progressive Matrices; BDI: Beck Depression Inventory.

To assess for potential differences in alexithymia among the three groups (HC, aMCI, naMCI), a series of ANCOVAs were conducted, with Group as the independent variable, the TAS scores as the dependent variables, and age and schooling as covariates. The Tukey test was chosen for *post hoc* analyses. Finally, linear correlations (Pearson’*r*) were performed to examine the relationships between TAS scores and cognitive functioning measures in the three different groups.

## Results

### Demographic, cognitive, and psychological characteristics

Mean, standard deviations of demographic, cognitive, and psychological variables are shown in Table 2.

ANOVAs results showed significant differences for age (df = 2,317, *F* = 11.8, *p* < 0.001, pη^2^ = 0.069), years of education (df = 2,317, *F* = 11.8, p < 0.001, pη^2^ = 0.069), ADL (df = 2,317, *F* = 5.22, *p* = 0.006, pη^2^ = 0.032), IADL (df = 2,317, *F* = 4.24, *p* = 0.015, pη^2^ = 0.026), MMSE scores (df = 2,317, *F* = 25.3, p < 0.001, pη^2^ = 0.137), RSPM scores (df = 2,317, *F* = 25.6, *p* < 0.001, pη^2^ = 0.139) and number of children (df = 2,317, *F* = 3.78, *p* = 0.024, pη^2^ = 0.023). Specifically, HC and naMCI were significantly younger than the aMCI group (HC = 60.3 v.s aMCI = 65.8; *p* < 0.001; naMCI = 61.6 vs. aMCI = 65.8; *p* = 0.004). HC group had significantly more years of education than both the naMCI (15.9 vs. 14.6; *p* = 0.036) and aMCI (15.9 vs. 13.2; p < 0.001).

RSPM scores were higher for the HC group than for both aMCI (37.2 vs. 30.9; *p* < 0.001) and naMCI (37.2 vs. 32.3; *p* < 0.001) groups. Moreover, considering the global level of cognitive functioning, individuals with aMCI had lower MMSE scores than individuals with naMCI (28.1 vs. 28.8; *p* = 0.002) and HC (28.1 vs. 29.3; *p* < 0.001); naMCI also had lower MMSE scores than HC, (*p* = 0.003). ADL was significantly lower in the aMCI group when compared with both HC (5.89 vs. 5.98; *p* = 0.012) and naMCI (5.89 vs. 6.0; *p* = 0.009). IADL follows the same pattern, being lower in the aMCI group than in HC (7.73 vs. 7.91; *p* = 0.025) and naMCI (7.73 vs. 7.94; *p* = 0.023). No differences emerged between groups for lifestyle habits and depression, except for the number of children: HC have significantly less children than aMCI (1.59 vs. 1.97; *p* = 0.028). Finally, the Chi-square test showed significant differences in the gender distribution (*χ*^2^ = 43.5, *p* = <0.001).

### Differences in alexithymia

Table 3 shows the differences in TAS-20 scores according to the Group. ANCOVAs were conducted, considering the Group as the independent variable, the TAS scores (TAS Total, TAS F1, TAS F2, TAS F3) as the dependent variables, and age and schooling as covariates.

**Table 3:** Mean (±SD) of TAS scores in the three groups of participants and ANCOVA results.

TAS scores	HC	aMCI	naMCI	*F*	*p*
	(*N* = 175)	(*N* = 64)	(*N* = 81)		
TAS Total	40.6 (11.99)	47.7 (12.85)	41.7 (10.13)	3.19	0.042
TAS F1	12.6 (5.07)	14.8 (6.48)	13.3 (5.35)	1.27	0.28
TAS F2	11.1 (4.76)	13.3 (4.73)	11.7 (4.43)	1.30	0.28
TAS F3	16.8 (5.58)	19.6 (5.02)	16.7 (4.90)	3.48	0.032

HC, healthy controls; aMCI: amnestic mild cognitive impairment; naMCI, non-amnestic mild cognitive impairment.

ANCOVAs showed significant differences in the TAS Total score (df = 2,317, *F* = 3.19, *p* = 0.042, pη^2^ = 0.020) and TAS F3 (df = 2,317, *F* = 3.48, *p* = 0.032, pη^2^ = 0.022). TAS Total is higher in aMCI than in HC (47.7 vs. 40.6; *p* = 0.047), while TAS F3 is higher in aMCI than naMCI (19.6 vs. 16.7; *p* = 0.029). No significant differences emerged between the naMCI and HC groups.

### Correlations between alexithymia score and cognitive functioning

Table 4 reports the correlations between TAS scores and cognitive performance in aMCI, naMCI and HC groups.

**Table 4:** Correlation matrix between cognitive functioning and TAS scores in HC, aMCI, and na MCI groups.

Cognitive assessment	aMCI				naMCI				HC			
	TAS	F1	F2	F3	TAS	F1	F2	F3	TAS	F1	F2	F3
MMSE	−0.17	−0.08	−0.21	−0.13	−0.26*	−0.14	0.03	−0.41***	0.03	0.05	0.07	−0.03
RSPM	−0.31*	−0.22	−0.30*	−0.23	−0.27*	−0.25*	−0.14	−0.16	−0.25**	−0.25 ***	−0.14	−0.18*
DSF	−0.21	−0.29*	−0.22	0.04	−0.20	−0.23*	0.04	−0.19	0.02	−0.08	0.007	0.12
RAVLT-I	−0.31*	−0.27*	−0.24	−0.21	0.001	0.07	−0.01	−0.06	−0.17*	−0.12	−0.1	−0.18*
RAVLT-D	−0.24	−0.16	−0.22	−0.20	0.06	0.18	0.01	−0.09	−0.13	−0.07	−0.1	−0.13
Babcock	0.08	0.05	−0.004	0.14	−0.14	−0.19	−0.09	0.004	−0.01	−0.03	0.007	0.001
IVM	0.13	0.13	0.08	0.10	−0.16	−0.08	−0.11	−0.14	−0.03	0.06	0.03	−0.14
FRD	−0.27*	−0.23	−0.31*	−0.09	0.11	−0.007	0.04	0.21	0.02	−0.02	0.05	0.03
PF	−0.22	−0.14	−0.25*	−0.14	−0.12	−0.08	−0.10	−0.07	−0.10	−0.07	−0.15	−0.02
SF	−0.16	−0.05	−0.25*	−0.10	−0.007	−0.005	−0.15	0.12	0.008	−0.08	−0.02	0.11
SC	−0.25	−0.27*	−0.14	−0.16	−0.09	−0.02	−0.09	−0.09	−0.03	−0.01	0.01	−0.08
AM	−0.30*	−0.20	−0.26*	−0.27*	−0.002	−0.10	0.05	0.07	0.008	−0.05	0.01	0.05
TMT-A	0.14	0.05	0.15	0.16	−0.05	0.15	−0.10	−0.18	0.06	0.12	0.02	−0.001
CDT	0.13	0.12	0.09	0.09	0.13	0.12	0.007	0.13	0.19*	0.06	0.10	0.26 ***
FRI	−0.33*	−0.36**	−0.22	−0.15	−0.06	0.03	−0.13	−0.04	−0.04	0.006	−0.04	−0.06
CD	−0.22	−0.11	−0.24	−0.20	−0.08	−0.09	−0.05	−0.02	−0.16*	−0.18*	−0.06	−0.13
CDL	−0.06	0.01	0.004	−0.17	−0.01	−0.16	−0.01	0.15	0.02	−0.04	0.04	0.05
DSB	−0.23	−0.20	−0.21	−0.12	0.005	−0.01	−0.05	0.07	−0.09	−0.11	−0.07	−0.04
TMT-B	0.14	0.13	0.03	0.17	0.22*	0.24*	0.03	0.15	0.006	0.07	−0.05	−0.01

*p < 0.05, **p < 0.01, ***p < 0.001.

MMSE: Mini-Mental State Examination; RSPM: Raven’s Standard Progressive Matrices; DSF: Digit Span Forward test, RAVLT-I: Rey Auditory Verbal Learning Test Immediate recall; RAVLT-D: Rey Auditory Verbal Learning Test Delayed recall; IVM: Immediate Visual Memory task; FRD: Rey-Osterrieth Complex Figure; SC: Sentence Construction test; PF: phonemic fluency; SF: semantic fluency; AM: Attentional Matrices; TMT-A: Trail Making Test-A; CDT: Clock Drawing Test; FRI: copy of the Rey-Osterrieth Complex figure; CD: copy drawings without landmarks; CDL: copy drawings with landmarks; DSB: Digit Span Backward test; TMT-B: Trail Making Test-B.

In the naMCI group, the MMSE correlated negatively with TAS Total and TAS F3, indicating that higher alexithymia was associated with poorer global cognitive functioning. Several other cognitive measures, including RSPM, DSF, and FRI, also showed negative correlations with TAS dimensions, particularly F1 and F2, suggesting that greater alexithymic traits were linked to reduced reasoning and attentional abilities. The TMT-B correlated positively with TAS Total and F1, reflecting a relationship between higher alexithymia and slower executive processing.

In the aMCI group, correlations were predominantly negative: RSPM, RAVLT-I, FRD, AM, and FRI all showed significant negative associations with TAS Total and/or its factors, suggesting that greater cognitive impairment was related to higher alexithymia, especially difficulties in identifying and describing feelings (F1 and F2).

In the HC group, the pattern was generally weaker; RSPM, RAVLT-I, and CD displayed small but significant negative correlations with TAS factors, whereas CDT showed a positive correlation with TAS Total and F3, indicating a weak association between alexithymic traits and specific visuospatial–executive abilities in healthy individuals.

## Discussion

The present study aimed at clarifying the contribution of alexithymia in cognitive aging. By comparing healthy participants and MCI participants, we aimed at exploring whether the association between deficits in emotional awareness and cognitive decline represents a continuum of normal aging or whether it is a marker of pathological processes. As hypothesized, we found that participants with MCI, and especially the amnestic subtype (aMCI), had significantly higher alexithymia scores compared to cognitively unimpaired controls even after controlling for age and education status. In addition, our findings show that within the MCI group, alexithymia was correlated negatively with several measures of cognitive performance, (i.e., memory, reasoning, language, and executive functions), whereas this was not the case for the healthy aging group. These findings point to a specific alexithymic-cognitive impairment link, that becomes apparent only when neurocognitive systems begin to deteriorate.

These findings are in keep with an emerging literature suggesting that alexithymia is more than a personality marker, and that it may represent an indicator of neural and functional breakdown. In fact, higher alexithymia scores have been observed in patients with MCI and Alzheimer’s disease compared to normal subjects (27, 28), and longitudinal findings indicate that emotion processing disturbance may be a pre- or co-morbid symptom of cognitive impairment (48).

The current findings add to the existing literature by showing that alexithymia may not only be typical of late life or to overt dementia, but it also occurs and it can be detected in middle-aged participants who are showing mild cognitive impairment, especially for the amnestic type. Importantly, the negative correlations between alexithymia and cognitive performance show that this association is not just limited to memory but to more general types of reasoning and overall cognition, suggesting shared neurocognitive processes.

More specifically, we found that MCI subjects, and particularly aMCI subtype, showed significantly higher alexithymia scores compared to cognitively unimpaired controls, after controlling for age and education. This finding provides new evidence that emotional awareness deficits are already detectable at earlier stages of cognitive impairment (i.e., the MCI stage) and may parallel or even contribute to early ToM and empathy decline. Considering that both alexithymia and ToM deficits involve difficulties in identifying, representing, and understanding emotional states—whether one’s own or others’—our findings point to a shared vulnerability of neural circuits subserving self-referential and social cognition. Accordingly, high alexithymia levels in aMCI may reflect an early breakdown of the mentalizing network, encompassing the medial prefrontal cortex, temporoparietal junction, and anterior cingulate cortex, regions critical for both ToM and emotional introspection. These findings align with the hypothesis that reduced emotional awareness (alexithymia) and impaired ToM represent two facets of a broader socio-emotional disintegration emerging in the prodromal stages of neurodegenerative disease.

Neurobiologically, alexithymia-cognitive impairment comorbidity is a sign of damage or impairment in overlapping neural networks that enable emotional processing and higher-order cognition. Neuroimaging studies have pointed toward the anterior cingulate cortex (ACC), insula, and medial prefrontal cortex as key regions in emotional awareness and interoception (21, 49). The same areas are significantly involved with executive control, attentional selection, and working memory. Structural damage or reduced connectivity within the salience network is consistently present in MCI and early Alzheimer’s disease (50, 51). Injury to these regions may thereby hamper both identification and emotional expression as well as the efficacy of cognitive control. Consistent with this evidence, Paradiso et al. (52) demonstrated that reduced right rostral cingulate volume was associated with increased alexithymia in older persons, and degeneration of the cingulate could be a neural substrate that links emotional unawareness with cognitive impairment.

The replicable relationship of alexithymia with memory function, particularly in amnestic MCI, also corroborates the hypothesis of shared vulnerability in fronto-limbic circuits (9). The hippocampus and the medial temporal structures that interface with it not only facilitate episodic memory but also interface with the amygdala and prefrontal cortex in the modulation of emotional memory and self-reflection (53). Functional deterioration in these networks can both impair memory retrieval as well as retrieval of internal representations of emotion. Consequently, as the neurodegenerative changes progress, the emotional life of the person may become impoverished, that is less differentiated and more externally oriented, as it is typical of the pensée opératoire delineated by Taylor et al. (16). In line with this proposal, our study shows that the amnestic MCI subgroup has significantly higher scores on the TAS-20 externally oriented thinking subscale, which is indicative of higher concrete, fact-bound thoughts and lower introspective awareness. This finding provides empirical evidence in support of the prediction that the cognitive inflexibility and reduced reflective function that are hallmarks of alexithymia can emerge together with mnemonic impairment, a result that is representative of shared neurocognitive mechanisms underlying both processes.

Most importantly, our findings also address the more general psychological and motivational mechanisms implicated in aging. Emotional theories of aging, for instance, Carstensen’s Socioemotional Selectivity Theory (54) and the Strength and Vulnerability Integration model (55), stipulate that older adults tend to develop greater emotional stability through prioritizing positive experiences and better affect regulation. However, these adaptive processes are based on intact cognitive and neural resources. As self-reflective capacity and executive control weaken, emotional regulation also diminishes, with greater rigidity, lower introspection, and reduced affect. Thus, the heightened alexithymia observed in MCI can suggest an atrophy of the adaptive emotional processes that allow for successful aging. Far from being an isolated deficit in emotion, alexithymia may represent a loss of cognitive–emotional integration (i.e., the diminished capacity of the brain to link internal states with concept representations and behavioral ends).

An alternative account calls on the interplay between alexithymia, interoceptive accuracy, and self-perception in late life. Xu et al. (56) recently demonstrated that higher alexithymia in older people is associated with a greater perceived age, reduced emotion regulation flexibility, and increased frailty. This suggests that alexithymic characteristics not only reflect unawareness of emotions but also altered sense of body and self and perhaps are a product of dysregulated interoceptive and autonomic processes. These processes may further exacerbate cognitive inefficiency through chronic stress, social isolation, or reduced engagement with affectively relevant stimuli. The blunting quality of alexithymia may thus augment the cognitive effects of aging by reducing environmental richness and possibilities for emotional learning.

An additional finding worth noting is that the aMCI group showed statistically lower ADL and IADL scores compared to HC and naMCI, although the absolute differences were small. While MCI is typically defined by preserved or minimally affected functional autonomy (5), subtle reductions—particularly in instrumental activities—have been reported in aMCI and may reflect very early functional vulnerability along the dementia continuum (i.e., subthreshold functional decline that is not yet clinically overt). In the present sample, this pattern could be linked to the aMCI clinical profile and/or to the older age and lower education of this group, which may contribute to modest functional inefficiencies despite overall independence. Notably, this subtle functional signal may also relate to alexithymia, especially externally oriented thinking, insofar as reduced introspective awareness and emotional self-monitoring could indirectly affect planning, self-initiation, and the organization required for complex instrumental tasks (e.g., medication management, finances). Future longitudinal studies combining informant-based and performance-based functional measures will be important to clarify whether these small ADL/IADL differences predict progression and whether alexithymia contributes independently to functional outcomes.

The clinical implications of the present findings are clear. First, alexithymia assessment may prove to be a useful adjunct to cognitive screening in neuropsychological or geriatric clinics. The identification of older adults with both cognitive inefficiencies and elevated alexithymia could mark individuals at elevated risk of progression to dementia. Second, interventions aimed at enhancing emotional awareness may re-establish some degree of emotional–cognitive coupling, promoting resilience and functional autonomy. There is evidence that targeted training in interoceptive awareness can not only enhance emotion regulation but executive functioning in older age (57), suggesting that rehabilitation of emotion may have secondary effects on cognition.

Despite these important implications, several limitations of the present study should be acknowledged. The cross-sectional approach limits causal inference: it is not possible to establish whether alexithymia causes cognitive deterioration, perhaps by diminishing social and emotional stimulation, or whether it arises as a result of declining cognitive control. Longitudinal and multimodal research combining cognitive [e.g., executive functions; (58)] behavioral [e.g., sleep quality and cognitive reserve; (59, 60)], neuroimaging, and neurobiological markers is required to disentangle these directions of influence. However, our findings provide clear hypotheses for future research. Moreover, although our sample was large and demographically representative, it consisted predominantly of women (68%) and was highly educated, which may limit the generalizability of the results to other populations with different socio-cultural or educational backgrounds. In addition, the study focused exclusively on alexithymia as an index of emotional regulation difficulties, without including other complementary measures of emotion regulation or coping styles, as well as ToM or mentalizing measures. Future research would also benefit from assessing different components of emotional processes and from integrating psychophysiological indices (e.g., Heart Rate Variability) to provide a more comprehensive understanding of the mechanisms linking emotional processes and cognitive functioning.

In conclusion, the present findings are in line with the proposal that cognitive and emotional aging are extremely interconnected processes with common underlying neural and psychological mechanisms. Although alexithymia is influenced by multiple interacting factors (e.g., developmental experiences and metacognitive capacities) and may show relative trait-like stability at the individual level, our findings suggest that elevated alexithymic features in MCI—particularly in aMCI—may represent a sensitive behavioral expression of diminished emotional–cognitive integration in the context of pathological aging. Importantly, because our comparison was confined to healthy older adults versus MCI subtypes, we do not draw conclusions about the effects of normal aging on alexithymia; addressing age-related change per se would require direct comparisons with younger healthy groups. Recognizing this phenomenon expands our understanding of the clinical phenotype of MCI and offers novel opportunities for early detection and intervention. Ultimately, integrating emotional and cognitive dimensions both in research and clinical practice may foster a more holistic and humane approach to aging.

## Data Availability

The raw data supporting the conclusions of this article will be made available by the authors, without undue reservation.
